# Epidemiology of mechanically ventilated patients treated in ICU and non-ICU settings in Japan: a retrospective database study

**DOI:** 10.1186/s13054-018-2250-3

**Published:** 2018-12-04

**Authors:** Yoshiaki Iwashita, Kazuto Yamashita, Hiroshi Ikai, Masamitsu Sanui, Hiroshi Imai, Yuichi Imanaka

**Affiliations:** 1 Emergency and Critical Care Center, Mie University Hospital, 2-174 Edobashi, Tsu, Mie Japan; 2 Department of Healthcare Economics and Quality Management, Kyoto University, Yoshida Konoe-cho, Sakyo-ku, Kyoto, 606-8501 Japan; 3 Jichi Medical University Saitama Medical Center, 1-847 Amanuma, Saitama, Saitama Japan

**Keywords:** ICU, Japan, Mechanically ventilated patient

## Abstract

**Background:**

In most countries, patients receiving mechanical ventilation (MV) are treated in intensive care units (ICUs). However, in some countries, including Japan, many patients on MV are not treated in ICUs. There are insufficient epidemiological data on these patients. Here, we sought to describe the epidemiology of patients on MV in Japan by comparing and contrasting patients on MV treated in ICUs and in non-ICU settings. A preliminary comparison of patient outcomes between ICU and non-ICU patients was a secondary objective.

**Methods:**

Data on adult patients receiving MV for at least 3 days in ICUs or non-ICU settings from April 2010 through March 2012 were obtained from the Quality Indicator/Improvement Project, a voluntary data-administration project covering more than 400 acute-care hospitals in Japan. We excluded patients with cancer-related diagnoses. Patient demographic data and the critical care provided were compared between groups.

**Results:**

Over the study period, 17,775 patients on MV were treated only in non-ICU settings, whereas 20,516 patients were treated at least once in ICUs (46.4% vs. 53.6%). Average age was higher in non-ICU patients than in ICU patients (72.8 vs. 70.2, *P* < 0.001). Mean number of ventilation days was greater in non-ICU patients (11.7 vs. 9.5, *P* < 0.001). Hospital mortality was higher in non-ICU patients (41.4% vs. 38.8%, *P* < 0.001). Standard critical care (e.g., arterial line placement, enteral nutrition, and stress-ulcer prevention) was provided significantly less often in non-ICU patients. Multivariate analysis showed that ICU admission significantly decreased hospital mortality (adjusted odds ratio 0.713, 95% CI 0.676 to 0.753).

**Conclusions:**

A large proportion of Japanese patients on MV were treated in non-ICU settings. Analysis of administrative data indicated preliminarily that hospital mortality rates in these patients were higher in non-ICU settings than in ICUs. Prospective analyses comparing non-ICU and ICU patients on MV by severity scoring are needed.

**Electronic supplementary material:**

The online version of this article (10.1186/s13054-018-2250-3) contains supplementary material, which is available to authorized users.

## Background

The numbers of patients requiring intensive care are increasing worldwide [1]. Many of these patients require mechanical ventilation (MV), and in a majority of countries they are treated exclusively in intensive care units (ICUs) [1, 2]. In some countries, including Japan, however, some patients on MV are treated in non-ICU settings. This is presumably because of a shortage of ICU beds in Japan compared with European countries [3–5], or shortages of trained nurses or critical care physicians. In Japan, because ICUs require official regulation, some hospitals have special units that are used to treat severely ill patients but are not officially certified as ICUs. The nurse-to-patient ratio in general wards is 1:7, but official ICUs are required to have a ratio of 1:2. It is difficult for small to medium-sized hospitals to employ enough nurses to meet this ratio. Therefore, some hospitals have non-certified ICUs. The organization of, and care in, these quasi-ICUs vary among hospitals, and patient selection depends on the region. There are no official criteria designating what kinds of patients should be treated in each type of ICU. If the quality of care provided in ICUs and these quasi-ICUs differs, then some patients may be receiving suboptimal care.

To the best of our knowledge, no epidemiological data on acute-phase patients on MV treated in non-ICU settings in Japan have been reported. Previously, we performed an attitude survey of physicians treating critically ill patients in hospitals without ICU facilities, and we found that 10% of physicians at these hospitals were treating acute-phase patients on MV approximately once a month [6]. However, because of a lack of quantitative epidemiological data in the survey, no conclusion was drawn on what proportion of patients were being treated in non-ICU settings. We consider that the feasibility and safety of treating patients on MV in non-ICU settings is worth investigating in a well-designed study.

Our primary objective here was to describe the epidemiology of patients on MV in Japan by comparing and contrasting these patients treated in ICUs and in non-ICU settings. We also compared the outcomes of ICU and non-ICU patients on MV by using the currently available dataset, as a basis for future studies.

## Methods

We compared patients on MV who were treated at least once in ICUs (ICU group) and those who never stayed in ICUs (non-ICU group). Patients whose treatment was started in non-ICU settings and who were then transferred to ICUs were counted in the ICU group, regardless of whether they started MV before or after ICU admission. “MV” includes both intubated MV and acute-phase non-invasive MV with partial arterial pressure of oxygen (PaO_2_) < 300 mmHg or partial arterial pressure of carbon dioxide (PaCO_2_) > 45 mmHg. “ICU” was defined here as “officially certified ICU” in accordance with Japanese government insurance policy. The criteria for an officially certified ICU include staffing of the unit with at least one physician in-house 24 hourly and with nurses at a ratio of one nurse to two patients; in addition, a certified ICU must be fully equipped with hardware to resuscitate critically ill patients. In Japan there are many quasi-ICU units that treat severely ill patients and function similar to ICUs but not officially certified. These quasi-ICUs were counted among non-ICUs.

Under the government insurance policy, ICU admission is allowed for patients who are in the following states: loss of consciousness, respiratory failure, cardiac failure, acute intoxication, shock, severe metabolic disorder, severe burns, after major surgery, post-resuscitation, and severe trauma. However, these are minimal requirements, and the actual indications depend on the facilities, the attending physicians, and the relative severity among ICU patients.

### Data source

The data were derived from the Quality Indicator/Improvement Project (QIP), which is an administrative database project covering more than 400 voluntary acute-care hospitals in Japan. The project is operated by Kyoto University. The dataset is based on a Japanese-government-operated national database (Diagnosis Procedure Combination (DPC)), which includes each patient’s demographic data and diagnosis (including the main reason for hospital admission, major complications, past major diagnoses, and co-morbidities).

### Target patients

**Fig. 1:**
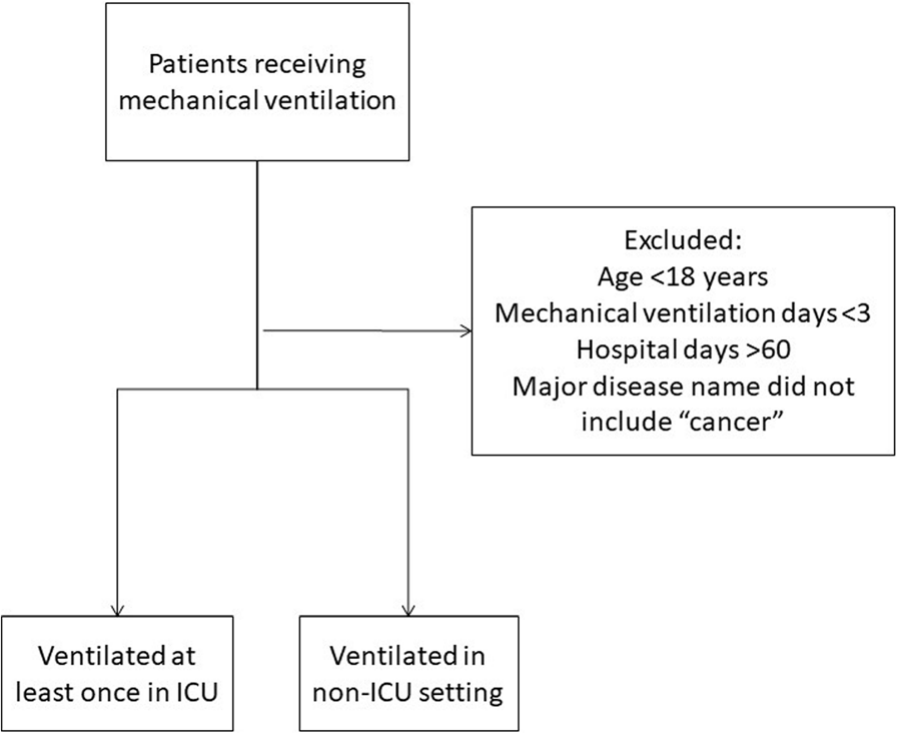
The selection of patients. Among patients who received invasive mechanical ventilation (MV), those who were younger than 18 years, those who received MV for fewer than 3 days, those who stayed in hospital for more than 60 days, and those who had a major diagnosis of cancer were excluded; 17,775 patients were ventilated only in non-ICU settings

We used patient data from the QIP database for the period from April 2010 through March 2012. Patient flow through the study is shown in Fig. 1. Patients who received MV for at least one period during their hospitalization and did not receive palliative care were eligible for inclusion in the study. Patients who were younger than 18 years, received MV for fewer than 3 days, had hospital stays of more than 60 days, or had cancer as their major disease were excluded. To prevent the data for analysis from being influenced by the inclusion of data from terminal-stage patients who were not candidates for ICU admission, we excluded patients who had cancer codes as their major current diagnoses but not in their past histories. Although exclusion of patients with end-stage non-cancerous conditions was not possible, the proportions of patients with severe heart failure (New York Heart Association stage 4) or severe respiratory failure (Hugh-Jones stage 4) were similar between patients in the ICU group and in non-ICU group (data not shown).

### Development of the prediction model

Model development was based on previous work done by Umegaki et al. [7]. Briefly, possible variables were derived by using methods developed by Umegaki et al., but with modifications. First, patients in the Umegaki model are all admitted to the ICU, whereas in our dataset not all patients were admitted to the ICU but all patients received MV. Among the demographic variables, age was defined as a continuous variable. Among the clinical factors, hospital admission categories were derived from the administrative data. Scheduled-admission patients were those admitted through a reservation provided during their previous hospital visit. Unscheduled or ambulance admissions were categorized as emergency admissions. Emergency admission included admissions direct to the hospital, by referral from other hospitals, or from the scene of the precipitating event or accident. Scheduled admission was used as a reference value for hospital admissions in the multivariate analysis. “Reasons for ICU admission” in the study by Umegaki et al. was replaced by “Reasons for starting MV.” “Patients who underwent surgery on the day of initiation of MV”, or “patients who began MV within 7 days after surgery” were replaced as “patients who underwent MV in relation to surgery.” Among these patients, those who underwent surgery on the day of hospital admission or the following day were defined as having had “emergency surgery,” whereas those who did not undergo emergency surgery were defined as having had “scheduled surgery.” All other patients were considered to have been admitted for medical reasons.

To determine the categories of primary diagnoses, we used the World Health Organization *International classification of diseases and related health problems 10th revision*, to translate our administrative data into diagnostic categories in accordance with the methods used by Umegaki et al. [7] (Additional file 1: Table S1). There were four categories of time between hospital admission and initiation of MV (days) (Table 1). Among the treatment categories, renal replacement therapy included continuous renal replacement therapy, intermittent renal replacement therapy, plasma absorption, and plasma exchange, but it excluded peritoneal dialysis, which is rarely used in patients on MV. Catecholamine treatments included administration of dopamine, dobutamine, noradrenaline, or adrenaline.

**Table 1:** Candidate variables used to develop the hospital mortality prediction model

Type	Candidate variable	Category
Demographic	Sex	Male; female
	Age	Continuous variable
Clinical factors	Hospital admission	Scheduled, emergency
	Reason for starting MV	After scheduled surgery; after emergency surgery; internal medicine patients
	Primary diagnosis	See Table 3
	Time between admission and initiation of MV	Immediate; 1 day; 2–4 days; > 4 days
	Renal replacement therapy	Yes = 1; no = 0
	Catecholamines	Yes = 1; no = 0

*MV* mechanical ventilation

The relationships between individual variables and hospital mortality were analyzed by the *t* test, and variables with *P* > 0.25 were excluded from subsequent analyses. The remaining variables were analyzed by multiple logistic regression using a backward stepwise selection method. The model was constructed using variables with *P* < 0.05. Significant variables and data on whether or not patients were admitted to the ICU were further analyzed by multiple logistic regression using a simultaneous method and adjusting for other variables to examine whether ICU admission was associated with the 28-day mortality rate.

Model performance was assessed in terms of discrimination and calibration. Model discrimination was assessed by using the area under the receiver operating characteristic (ROC) curve. The calibration was assessed using Hosmer–Lemeshow contingency χ^2^ statistics and a calibration plot curve. The calibration plot curve was described using R. All other statistical analyses were performed using SPSS software, version 23 (IBM Inc., Tokyo, Japan.)

## Results

### Epidemiological data

**Fig. 2:**
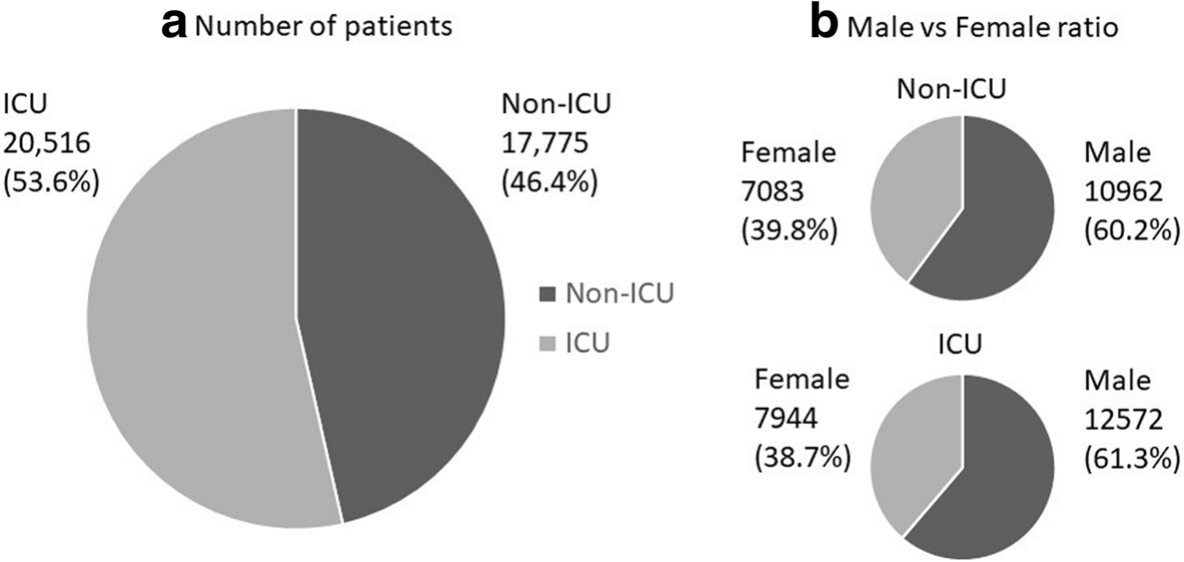
**a** Number of patients treated in ICU and non-ICU setting. Nearly half of acute-phase patients on mechanical ventilation (MV) were treated in non-ICU settings. **b** Male versus female ratio of patients on MV treated in ICU and non-ICU settings

**Fig. 3:**
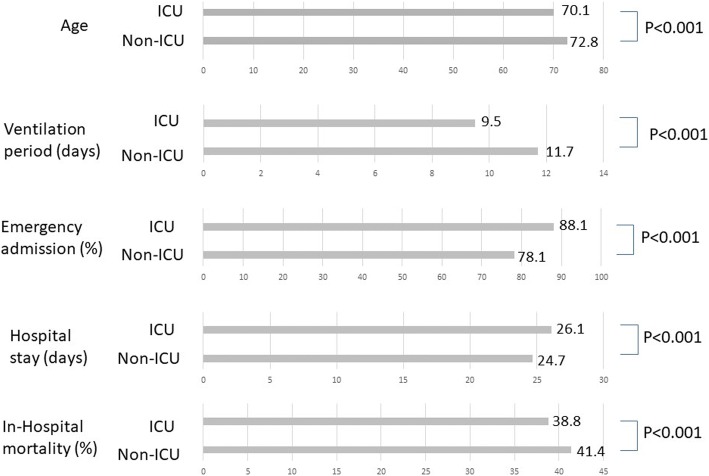
Comparison of ICU and non-ICU patients. Non-ICU patients were significantly older. Ventilation period was longer in non-ICU and in-hospital mortality was higher in non-ICU patients, while hospital stay was significantly higher in ICU patients

In the study period, 17,775 patients with MV (46.4%) were exclusively treated in the general ward, whereas 20,516 patients with MV (53.6%) were treated for at least one period in the ICU (Fig. 2). The percentage of men and the percentage of emergency admissions were significantly higher in ICU patients (*P* = 0.025, *P* < 0.001, respectively). Univariate comparisons of ICU and non-ICU patients are shown in Fig. 3. Average age was significantly higher in non-ICU patients than in ICU patients (72.8 vs. 70.1 years; *P* < 0.001). The mean number of ventilation days was significantly greater in the non-ICU group than in the ICU group (11.7 vs. 9.5 days; *P* < 0.001). Overall hospital mortality was significantly higher in non-ICU patients than in ICU patients (7353 (41.4%) vs. 7963 (38.8%); *P* < 0.001).

### Treatment options

**Fig. 4:**
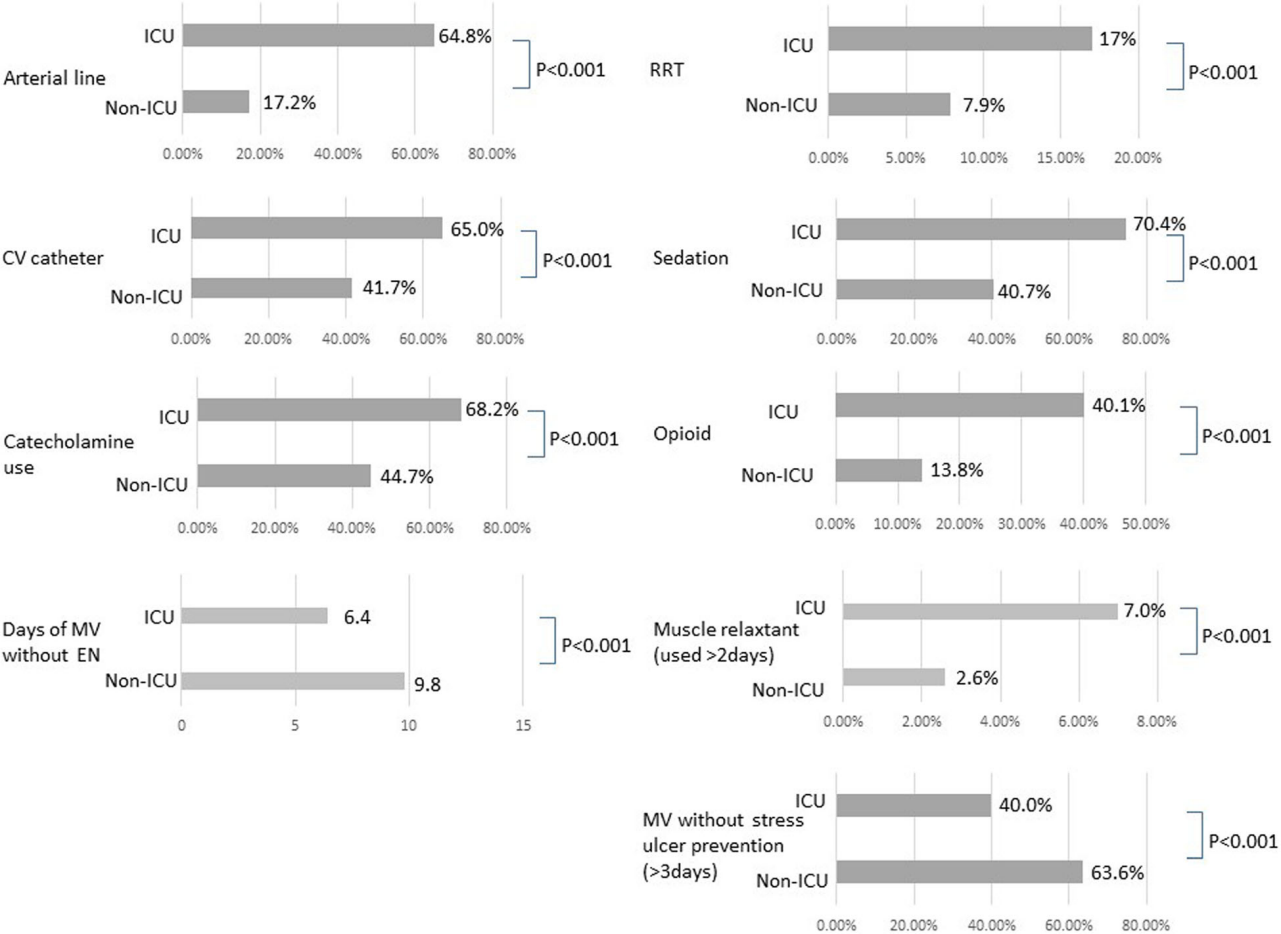
Treatments frequently applied to patients on mechanical ventilation (MV) in the ICU. Invasive lines were used in significantly fewer non-ICU patients than in ICU patients. There was significantly less use of catecholamines and renal replacement in non-ICU patients. There was shorter duration of enteral nutrition (EN) and less frequent use of stress-ulcer prophylaxis in the non-ICU group. CV, central vein; RRT, renal replacement therapy

Treatments frequently applied to patients on MV in the ICU are listed in Fig. 4. Invasive lines (arterial lines and central venous catheters) were used in significantly fewer non-ICU patients than ICU patients (arterial line, 17.2% vs. 64.8%, *P* < 0.001; central venous catheter: 41.7% vs. 65.0%, *P* < 0.001). There was significantly less use of catecholamines and renal replacement in non-ICU patients (catecholamines, 44.7% vs. 68.2%, *P* < 0.001; renal replacement, 7.9% vs. 17.0%, *P* < 0.001). There was shorter durations of enteral nutrition (EN) and less frequent use of stress-ulcer prophylaxis in the non-ICU group (average days of MV without EN, 9.8 vs. 6.4, *P* < 0.001; MV without stress-ulcer prevention for more than 3 days, 63.6% vs. 40.0%, *P* < 0.001).

### Major diagnoses

Numbers and percentages of major diagnoses are listed in Table 2. Heart failure was the most frequent reason for MV in either group (14.0% in the non-ICU group and 12.3% in the ICU group). Diseases requiring surgical interventions, including interventional radiology—such as aortic dissection, acute myocardial infarction, and subarachnoid hemorrhage—were frequently managed in ICUs. Diseases common in elderly patients, such as aspiration pneumonia, were more frequently managed in non-ICU settings.

**Table 2:** The number and percentage of major diagnoses

	Total (38,291)		Non-ICU (17,775)		ICU (20,516)	
Heart failure	5013	(13.1%)	2489	(14.0%)	2524	(12.3%)
Respiratory failure	1818	(4.7%)	1256	(7.1%)	562	(2.7%)
AMI	1813	(4.7%)	473	(2.7%)	1340	(6.5%)
Aspiration pneumonia	1696	(4.4%)	1005	(5.7%)	691	(3.4%)
Aortic dissection	1635	(4.3%)	211	(1.2%)	1424	(6.9%)
Sepsis	1434	(3.7%)	506	(2.8%)	928	(4.5%)
SAH	1356	(3.5%)	516	(2.9%)	840	(4.1%)
Cerebral hemorrhage	1316	(3.4%)	538	(3.0%)	778	(3.8%)
Interstitial pneumonia	1305	(3.4%)	843	(4.7%)	462	(2.3%)
Pneumonia	1289	(3.4%)	834	(4.7%)	455	(2.2%)
Other diagnoses	19,619	(51.2%)	9557	(53.8%)	10,512	(51.2%)

*AMI* acute myocardial infarction, *SAH* subarachnoid hemorrhage

### Epidemiological data according to diagnosis

To define the characteristics of ICU and non-ICU patients, we further analyzed three major diseases: heart failure, sepsis, and aspiration pneumonia (Table 3). We chose these diseases because they are treated in any type of hospital (i.e., they do not usually require surgical intervention). The number of patients with heart failure was similar between the ICU and non-ICU groups, whereas patients with sepsis were more frequently treated in ICUs and patients with aspiration pneumonia were more frequently treated in non-ICU settings. Regardless of the diagnosis, in-hospital mortality rates were significantly higher in non-ICU groups (heart failure, *P* = 0.045; sepsis, *P* = 0.001; aspiration pneumonia, *P* < 0.001). The age of patients treated in non-ICU settings was significantly higher in patients with heart failure (*P* = 0.037) and aspiration pneumonia (*P* < 0.001) but not in patients with sepsis (*P* = 0.134). In patients with sepsis, use of catecholamines (*P* < 0.001) and renal replacement (*P* < 0.001) therapies were more frequent in the ICU group. These data suggest that septic ventilated patients have worse outcomes in non-ICU settings, regardless of the fact that their disease may be less severe and despite their similar ages to ICU patients.

**Table 3:** Comparison of non-ICU and ICU patients according to three different diagnoses

	Heart failure					Sepsis					Aspiration pneumonia				
	Non-ICU		ICU		*P*	Non-ICU		ICU		*P*	Non-ICU		ICU		*P*
Number of patients	2489		2524			506		928			1005		691		
Age	76.6		75.9		0.037	74.0		72.9		0.134	79.8		75.5		< 0.001
Male	1341	53.9%	1477	58.5%	0.001	302	59.7%	565	60.9%	0.692	626	62.3%	428	61.9%	0.919
Emergency admission	2119	85.1%	2409	95.4%	< 0.001	445	87.9%	854	92.0%	0.014	904	90.0%	660	95.5%	< 0.001
Hospital stay, days	26.9		26.5		< 0.001	26.5		26.6		0.978	27.8		26.3		0.061
Number of days of ventilation	11.0		8.3		< 0.001	11.6		11.3		0.658	12.3		10.6		0.001
In-hospital deaths	608	24.4%	556	22.0%	0.045	343	67.8%	546	58.8%	0.001	542	53.9%	253	36.6%	< 0.001
Treatments															
Arterial line	300	12.1%	1226	48.6%	< 0.001	188	37.2%	721	77.7%	< 0.001	61	6.1%	295	42.7%	< 0.001
Central venous catheter	796	32.0%	1293	51.2%	< 0.001	421	83.2%	852	91.8%	< 0.001	460	45.8%	347	50.2%	0.75
Catecholamines	1115	44.8%	1547	61.3%	< 0.001	431	85.2%	858	92.5%	< 0.001	506	50.3%	370	53.5%	0.199
Renal replacement therapy	162	6.5%	323	12.8%	< 0.001	171	33.8%	436	47.0%	< 0.001	24	2.4%	25	3.6%	0.143
Sedation	715	28.7%	1361	53.9%	< 0.001	350	69.2%	756	81.5%	< 0.001	416	41.4%	446	64.5%	< 0.001
Opioids	207	8.3%	514	20.4%	< 0.001	135	26.7%	394	42.5%	< 0.001	42	4.2%	118	17.1%	< 0.001
Muscle relaxant	96	3.9%	383	15.2%	< 0.001	159	31.4%	380	40.9%	< 0.001	58	5.8%	137	19.8%	< 0.001
Enteral nutrition	244	9.8%	683	27.1%	< 0.001	149	29.4%	534	57.5%	< 0.001	334	33.2%	372	53.8%	< 0.001
Mechanical ventilation without stress ulcer prevention (≥ 3 days)	1785	71.7%	1352	53.6%	< 0.001	233	46.0%	328	35.3%	< 0.001	671	66.8%	364	52.7%	< 0.001

### Outcomes in ICU and non-ICU patients

We performed multivariate analysis to clarify further whether management in the ICU was associated with a decrease in hospital mortality rates. We developed a logistic regression model to eliminate possible confounding factors. Table 1 and Additional file 1: Table S1 show the variables that could potentially influence hospital mortality rates. These variables were chosen from previous studies [7–9]. The results of the multivariate analyses are shown in Table 4. ICU admission was significantly associated with a decrease in the hospital mortality rate (OR 0.713, 95% CI 0.676 to 0.753).

**Table 4:** Results of multivariable logistic regression analysis of hospital mortality in mechanically ventilated patients

	Exp(B)	95% CI	
Age	1.032	1.030	1.034
Admission category (emergency admission)	1.663	1.540	1.796
Reason for MV start			
After scheduled surgery	0.247	0.222	0.276
After emergency surgery	0.465	0.430	0.503
Anemia	6.582	2.212	19.592
Aplastic anemia	6.290	1.178	33.594
Bacterial sepsis	1.468	1.261	1.708
Cardiac arrest	6.217	5.509	7.017
Cardiac failure	0.470	0.432	0.512
Drug poisoning	0.438	0.256	0.750
Enteritis/colitis	3.172	0.750	13.412
Fluid and electrolyte disorder	1.512	1.092	2.096
Fungal sepsis	5.453	2.493	11.928
Gastrointestinal investigation	1.959	1.032	3.720
Head injury	5.945	4.978	7.100
Interstitial lung disease	3.683	3.258	4.163
Intracranial hemorrhage	10.134	9.104	11.281
Liver disease	3.790	2.462	5.833
Lower-limb trauma	1.454	1.053	2.010
Malignancy other	4.450	2.376	8.333
Myocardial ischemia	0.837	0.756	0.928
Other CNS disease	4.928	4.006	6.061
Penetrating trauma	3.238	2.001	5.241
Pneumoconiosis	1.622	1.421	1.851
Pneumonia	1.741	1.579	1.921
Protozoal sepsis	2.641	1.240	5.623
Respiratory failure	1.153	1.035	1.285
Stroke or cerebrovascular accident	3.405	2.819	4.112
Time from admission to MV start			
1 day	1.109	1.030	1.194
2–4 days	1.721	1.592	1.859
> 4 days	3.528	3.273	3.802
Renal replacement therapy	3.567	3.313	3.840
Catecholamine	5.161	4.876	5.462
ICU admission	0.713	0.676	0.753
Constant	0.010		

*CNS* central nervous system, *MV* mechanical ventilation

The discriminatory ability of the model was assessed by the area under the ROC, and the calibration was assessed by the Hosmer–Lemeshow test and a calibration plot curve (Additional file 2: Figure S1). The discrimination by the model in the area under the ROC was 0.818. Although the Hosmer–Lemeshow χ^2^ statistics showed a significant difference between the predicted and observed risk of mortality in our dataset, on the calibration plot curve our prediction model seemed to be well-matched to the observed risk of hospital death.

## Discussion

To our knowledge, this is the first survey of the characteristics and outcomes of patients with MV in non-certified ICU settings in Japan, compared with those in official ICUs. Surprisingly, in Japan, 46.4% of patients on MV were treated exclusively in non-ICU settings, including in quasi-ICUs and general wards. Patients treated in non-ICU settings were older, were less likely to have had emergency admissions, and had diagnostic characteristics that differed from those of patients in the ICU. Also, patients who needed surgical intervention, such as acute myocardial infarction (AMI), aortic dissection and SAH were more likely to be treated in the ICU. The overall number of ventilation days and hospital mortality rates were higher in non-ICU patients. In patients with sepsis there was no significant difference in age between groups, but there was greater mortality in non-ICU patients, who also received fewer critical care interventions. This suggests that there may be patients who are candidates for ICU treatment but who are being treated in non-ICU settings. This possibility is further supported by the results of the comparison of mortality rates between the ICU and non-ICU settings. The mortality rate adjusted by the administrative data was higher in the non-ICU group. Standard critical care, including EN and stress-ulcer prophylaxis, was less likely to be administered in non-ICU patients. These data may reflect the results of sustained therapy in terminally ill patients or patients with less severe disease who enter non-ICU settings; nevertheless, the reduced likelihood of receiving such treatments could lead to worse recovery outcomes in critically ill patients.

Ideally, critically ill patients requiring life-sustaining interventions should be treated in the ICU. However, one study showed that 16–51% of patients who need critical care are refused ICU admission because of limited resources [10]. In a large number of countries, despite limited ICU beds, the majority of acute patients on MV are still treated in ICUs [4], whereas in Japan patients on MV may be treated in non-ICU settings. We defined an ICU here as an “officially certified ICU.” Some of the quasi-ICU facilities in our study may well have been similar to ICUs. However, because admission fees are higher in certified ICUs, it is not likely that any certifiable facility would not obtain certification. Therefore, quasi-ICU units are likely to be inferior to certified ICUs in some regard.

Two reports—from Hong Kong and Israel—have compared patients on MV treated in ICUs with those treated in general wards [11, 12]. The Hong Kong study compared the mortality rates of patients on MV in non-ICUs with expected mortality rates. The Israeli study compared patients on MV treated in ICUs and those treated in non-ICUs. Both studies showed increase in mortality rates in non-ICU settings. However, both studies were relatively small, single-center studies, with a high institutional bias. In contrast, our results were derived from data on more than 38,000 patients from across Japan; this large number may help markedly to eliminate potential institutional biases.

Our results showed lower mortality in patients on MV if treated in ICUs. In contrast, previous studies have shown that mortality rates in patients with acute lung injury or catheter-related bloodstream infection have not decreased with patient treatment in the ICU [13–15]. In fact, patients with either of these conditions may not necessarily need critical care if their condition does not deteriorate. However, if critically ill patients on MV stay in the ICU, they may benefit from close monitoring and standard critical care (e.g., arterial line placement and stress-ulcer prevention) supplied by sufficient numbers of staff and specialized personnel (e.g., board-certified physicians and specialist nurses). Our results show that EN, stress-ulcer prevention, and arterial lines were used significantly less often in non-ICU patients with MV. Also, the Israeli study showed that the numbers of ventilator-setting changes and blood gas analyses were higher in ICU patients and the numbers of endotracheal-tube-related adverse events were higher in non-ICU patients [12]. These results indicate that patients with MV may be treated optimally in the ICU.

However, even for patients treated in the ICU, the management and outcomes may differ among institutions. In the USA, a comparison between patients on MV treated in rural hospitals with relatively limited resources and those treated in referral hospitals with better resources identified higher mortality rates in rural hospitals [16]. Also in Japan, ICUs certified by academic societies have better patient outcomes than those not certified [17]. Further research focusing on differences in intensive care quality among institutions is warranted.

Our study had several limitations. The biggest was that the QIP database does not include commonly used severity scores such as Acute Physiology and Chronic Health Evaluation (APACHE) II scores or Sequential Organ Failure Assessment (SOFA). Therefore, the difference in mortality rates between the groups might have been affected by differences in patient disease severity. For instance, the non-ICU group may have included more patients in the terminal state. However, we did eliminate patients with terminal-stage cancer from the target population. The numbers of patients with New York Heart Association stage-4 heart failure in the two groups were similar (data not shown). We also performed multivariate data analysis using the administrative data, revealing that ICU admission was significantly associated with a decrease in mortality. The validity of similar data adjustment using administrative data was previously shown to have a similar effect on APACHE II or SOFA scores [7–9]. Another limitation is that we were unable to evaluate ventilator settings and ventilator-induced adverse events, because our database did not include information on ventilator management. In hospitals without ICU facilities, there is potential for a delay in the introduction of current standards of care and new technology in MV owing to a potential lack of awareness of updates in the rapidly changing field of critical care. A center-related effect may also have affected the results: some hospitals may have larger numbers of non-ICU patients than others. Identifying these effects and the reasons behind them may further elucidate Japanese critical care epidemiology, but it would likely be difficult to do this with the current dataset. Future research, including research into ventilator management, may be of interest. Note also that the use of central venous lines, arterial lines, and catecholamines may not only be a consequence of being in an ICU or non-ICU setting but also a prognostic factor. Some facilities may not allow the use of these tools in non-ICU settings, regardless of the patient’s condition. This may result in delays in resuscitation or in awareness of hypoperfusion. For this reason, we think that the use of such items may be a prognostic factor as well. Moreover, co-morbidities might be important factors in predicting prognosis. However, we used the model of Umegaki et al. [7], which has already been shown to have sufficient prognostic value without the need to add co-morbidities.

## Conclusions

We compared care and outcomes in patients on MV in non-ICU and ICU settings using a large Japanese database. A large proportion of patients on MV were treated in non-ICU settings. The mortality rate in patients with MV was higher in non-ICU settings than in ICU settings, even after multivariate analysis. Considering that the criteria used to select patients for entry to ICUs is unclear, there may be a group of patients who may benefit from being treated in ICUs but are treated in non-ICU settings. Future research that includes clinically relevant data on patients with MV is needed to identify the factors that can help improve outcomes in these patients.

## Additional files

Additional file 1: **Table S1.** Candidate disease variables associated with hospital mortality rates, and their World Health Organization *International classification of diseases and related health problems 10th revision.* (DOCX 29 kb)

Additional file 2: **Figure S1.** Calibration plot of the logistic regression model. Hospital mortality risk values predicted by our model matched the observed risk values. (JPG 34 kb)

## Abbreviations

AMI: Acute myocardial infarction
APACHE: Acute Physiology and Chronic Health Evaluation
CNS: Central nervous system
CV: Central vein
DPC: Diagnosis procedure combination
EN: Enteral nutrition
ICU: Intensive care unit
MV: Mechanical ventilation
QIP: Quality indicator/improvement project
ROC: Receiver operating characteristic
RRT: Renal replacement therapy
SAH: Subarachnoid hemorrhage
SOFA: Sequential Organ Failure Assessment
