# Supplementary material for: Epidemiology of mechanically ventilated patients treated in ICU and non-ICU settings in Japan: a retrospective database study
Source: Crit Care. 2018 Dec 4;22:329. doi: 10.1186/s13054-018-2250-3 (PMC6280379; doi:10.1186/s13054-018-2250-3)
Supplement: Supplementary file 1 — Table S1. Candidate disease variables associated with hospital mortality rates, and their World Health Organization International classification of diseases and related health problems 10th revision. (DOCX 29 kb) [file 13054_2018_2250_MOESM1_ESM.docx]

Table S1. Candidate disease variables associated with hospital mortality rates, and their World Health Organization International Classification of Diseases (10th revision) categories

| Disease name | ICD-10 |
| --- | --- |
| Anemia | D5 |
| Aplastic anemia | D60–61 |
| Bacterial sepsis | A4 |
| Breast cancer | C5 |
| Cardiac arrest | I46 |
| Cardiac arrhythmias | I47–49 |
| Cardiac failure | I50 |
| CNS malignancy | C69–72 |
| Chronic obstructive pulmonary disease | J40–44 |
| Drug poisoning | T36–50 |
| Enteritis or colitis | K50–52 |
| Environmental disease | T66–78 |
| Epilepsy | G40 |
| Fluid and electrolyte disorders | E86–88 |
| Fungal sepsis | B35–49 |
| Gastrointestinal investigation | R1 |
| Hemopoietic malignancy | C81–96 |
| Hemorrhagic shock | R57–58 |
| Head injury | S0 |
| Interstitial lung disease | J8 |
| Intracranial hemorrhage | I60–62 |
| Ischemic bowel | K55 |
| Liver disease | K7 |
| Lower-limb trauma | S7 |
| Lung malignancy | C3 |
| Malabsorption | K90 |
| Malignancy – other | D37–48 |
| Myocardial ischemia | I20–25 |
| Other cerebrovascular disease | I65–69 |
| Other CNS disease | G9 |
| Other intestinal disease | K63 |
| Pancreatic cancer | C25 |
| Penetrating trauma | T15–19 |
| Pneumoconiosis | J60–70 |
| Pneumonia | J12–18 |
| Protozoal sepsis | B50–64 |
| Pulmonary vascular disease | I26–28 |
| Renal failure | N17–19 |
| Respiratory failure | J96 |
| Secondary malignancy | C76–80 |
| Stroke or cerebrovascular accident | I63–64 |
| Type 2 diabetes | E11 |
