# Supplementary figures and images for: Epidemiology of mechanically ventilated patients treated in ICU and non-ICU settings in Japan: a retrospective database study
Source: Crit Care. 2018 Dec 4;22:329. doi: 10.1186/s13054-018-2250-3 (PMC6280379; doi:10.1186/s13054-018-2250-3)

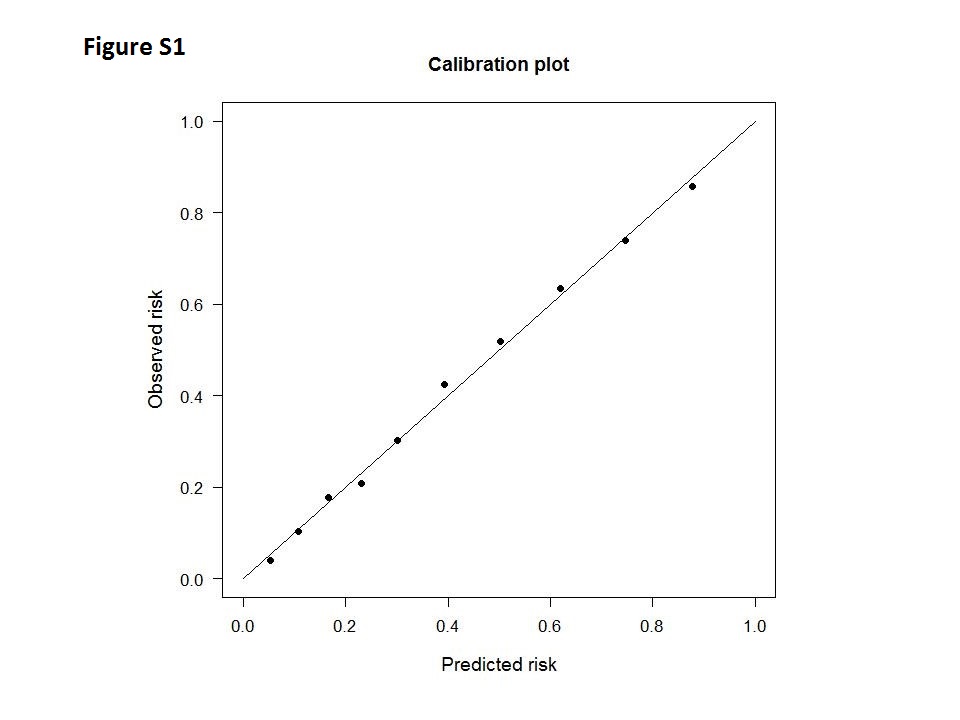

Supplement: Supplementary file 2 — Figure S1. Calibration plot of the logistic regression model. Hospital mortality risk values predicted by our model matched the observed risk values. (JPG 34 kb) [file 13054_2018_2250_MOESM2_ESM.jpg]
